# Semantic Memory and Lexical Availability in Parkinson’s Disease: A Statistical Learning Study

**DOI:** 10.3389/fnagi.2021.697065

**Published:** 2021-07-30

**Authors:** Juan F. Cardona, Johan S. Grisales-Cardenas, Catalina Trujillo-Llano, Jesús A. Diazgranados, Hugo F. Urquina, Sebastián Cardona, Alejandra Torres, Liliana A. Torres, Lina M. Gonzalez, Tania Jaramillo, Judith Cediel, Nelcy Oñate-Cadena, Geral Mateus-Ferro, Fernando Marmolejo-Ramos

**Affiliations:** ^1^Facultad de Psicología, Universidad del Valle, Santiago de Cali, Colombia; ^2^Centro de Investigación en Neurociencia Clínica y Comportamental (CINCCO), Universidad del Valle, Santiago de Cali, Colombia; ^3^Centro Médico de Atención Neurológica “Neurólogos de Occidente”, Santiago de Cali, Colombia; ^4^Instituto Neurológico del Pacifico, Santiago de Cali, Colombia; ^5^Departamento de Lenguas, Universidad Pedagógica Nacional, Bogotá, Colombia; ^6^Centre for Change and Complexity in Learning, University of South Australia, Adelaide, SA, Australia

**Keywords:** Parkinson’s disease, semantic memory, verbal fluency, lexical availability, embodied cognition

## Abstract

Parkinson’s disease (PD) is a neurodegenerative disorder that causes a progressive impairment in motor and cognitive functions. Although semantic fluency deficits have been described in PD, more specific semantic memory (SM) and lexical availability (LA) domains have not been previously addressed. Here, we aimed to characterize the cognitive performance of PD patients in a set of SM and LA measures and determine the smallest set of neuropsychological (lexical, semantic, or executive) variables that most accurately classify groups. Thirty early-stage non-demented PD patients (age 35–75, 10 females) and thirty healthy controls (age 36–76, 12 females) were assessed via general cognitive, SM [three subtests of the CaGi battery including living (i.e., elephant) and non-living things (i.e., fork)], and LA (eliciting words from 10 semantic categories related to everyday life) measures. Results showed that PD patients performed lower than controls in two SM global scores (picture naming and naming in response to an oral description). This impairment was particularly pronounced in the non-living things subscale. Also, the number of words in the LA measure was inferior in PD patients than controls, in both larger and smaller semantic fields, showing a more inadequate recall strategy. Notably, the classification algorithms indicated that the SM task had high classification accuracy. In particular, the denomination of non-living things had a classification accuracy of ∼80%. These results suggest that frontostriatal deterioration in PD leads to search strategy deficits in SF and the potential disruption in semantic categorization. These findings are consistent with the embodied view of cognition.

## Introduction

How are concepts stored in our minds? Since the conceptual framework of Collins and Quillian (1969), theoretical approaches have emerged in the field of semantic memory (SM) (Tulving, 1972; Caramazza and Hillis, 1991; Ullman, 2001, 2004; Caramazza and Mahon, 2006; Gainotti, 2015; Kumar, 2021). Neuroimaging studies have highlighted the involvement of modality-specific (sensory, cognitive, and motor) and multimodal neural circuits distributed in the frontal, temporal, and parietal cortex (Simons and Spiers, 2003; Binder and Desai, 2011; Quiroga, 2012). These findings have made it possible to identify a widely distributed cortical network associated with declarative memory.

Semantic fluency (SF) (Bousfield and Sedgewick, 1944) has been a classic SM measure in clinical and experimental neuropsychology. SF is the ability to identify specific categories (i.e., concepts, items, names, and objects) through association in a long-term memory store (Capitani et al., 2003; Robinson et al., 2012). Lexical availability (LA) tasks, which are typically used to identify the potential lexicon that a speaker possesses (of a mother tongue or a foreign language), have essentially the same features of the semantic fluency task (Hernández-Muñoz et al., 2014) with the critical addition of having defined categories (semantic fields) that are relevant to the everyday life of a speaking community, making them especially useful for SF studies.

The critical role of frontal and temporal cortical areas in SF performance has been well-studied. Neuropsychological studies have made it possible to partially identify the neural substrates of the conceptual organization and SM impairments’ characteristics. Patients with frontal damage have shown monitoring deficits and poor strategies during the retrieval process (Warrington and Shallice, 1984; Baldo and Shimamura, 1998; Stuss et al., 1998; Troyer et al., 1998; Schwartz and Baldo, 2001; Fuster, 2008; Squire, 2009; Squire and Wixted, 2011; Robinson et al., 2012). These deficits have also been reported in the behavioral variant of frontotemporal dementia (bvFTD) (Burgess and Shallice, 1997; Mayr, 2002; Reverberi et al., 2006, 2014; Possin et al., 2013). Furthermore, temporal lobe damage has been associated with worse performance on semantic fluency tasks (Campanella et al., 2010). Similar findings have been reported in the semantic variant of primary progressive aphasia (sv-PPA) (Hodges et al., 1992; Catricalà et al., 2014; Reverberi et al., 2014; Migliaccio et al., 2016).

Semantic categorization (SC) is a fundamental ability to recognize and classify an object. Indeed, identifying whether a stimulus is a living or non-living object allows us to make inferences and predictions about its behavior and its relationship with the context (Binder and Desai, 2011). The dissociation between semantic categories has been previously addressed. In their seminal work, Damasio and Tranel (1993) reported the dissociated naming performance for objects and verbs in three patients with predominantly frontal or temporal lesions. Recently, the study of neurodegenerative motor disorders also supports the differential role of frontal (motor and premotor) areas in action-verb processing (De Renzi and Di Pellegrino, 1995; Bak et al., 2001, 2006). A relevant dissociation deficit found in PD patients is that of manipulated vs. non-manipulated object naming. These patients perform lower (i.e., accuracy of responses) than controls when naming manipulated objects, but their performance is similar when naming non-manipulated objects (Johari et al., 2019). Notably, response times in manipulated object naming tasks seem to improve in early PD patients receiving both pharmacological and subthalamic DBS treatment (but not pharmacological treatment alone), contrary to non-manipulated object naming. However, accuracy seems to improve for neither type of object (Phillips et al., 2012).

SM is not limited to cortical regions but also extends into the subcortical areas. Currently, it is recognized the role of the basal ganglia in SM (Copland, 2003; Crosson et al., 2003; Longworth et al., 2005; Cardona et al., 2013). Several studies have shown that SM is impaired in Parkinson’s disease (PD) patients (Henry and Crawford, 2004; Kudlicka et al., 2011; Angwin et al., 2017). However, the cortico-subcortical circuits’ role in PD in categorizing and storing information in the living vs. non-living categories is not clear.

The purpose of the present study was to characterize the cognitive performance of PD patients using a comprehensive set of LA and SM tasks that included living/non-living categories. Importantly, this study aimed to determine the smallest set of neuropsychological (executive, semantic, or lexical) variables that could better classify participants as being PD or control with high accuracy. To our knowledge, the current research is the first to study LA to explore semantic fluency in PD.

## Materials and Methods

### Participants

The study comprised thirty early-stage non-demented PD patients and thirty healthy controls (all right-handed). PD patients’ clinical diagnosis was established by an expert neurologist (J.D) following the United Kingdom PD Society Brain Bank Criteria (Hughes et al., 1992). Their motor symptoms and disease stage were assessed using the Unified Parkinson’s Disease Rating Scale (UPDRS) (Fahn and Elton, 1987) and the Hoehn and Yahr scale (H&Y) (Hoehn and Yahr, 1967), respectively. All patients were receiving antiparkinsonian therapy and evaluated during the “on” phase of their medication. Control subjects were matched for age, sex, and years of education (see Table 1).

**TABLE 1:** Demographic, clinical, and neuropsychological data.

		**PD patients (*n* = 30)**	**Controls (*n* = 30)**	**PD vs. controls**			
		**Median (±MAD)**	**Median (±MAD)**	**γ**	***df***	***p***	**ξ**
**Demographics**							
Age (years)^a^		67 (6.67)	62.50 (8.15)	1.49	33.85	0.14	0.26
Sex (M: F)^b^		20:10	18:12	0.29		0.59	
Education (years)^a^		11 (4.45)	11 (2.97)	0.93	33.86	0.36	0.18
**Clinical assessment**							
Years since diagnosis^a^		2.8 (1.3)	–	–			
H&Y^a^		1.1 (0.3)	–	–			
UPDRS III^a^		25.47 (7.99)	–	–			
GDS^a^		1.50 (2.22)	2 (1.48)	0.83	33.93	0.41	0.17
IADL^a^		8 (0)	8 (0)	–^c^			
ADL^a^		100 (0)	100 (0)	–^c^			
**Cognitive measures**							
ACE-R^a^		92 (4.45)	92.50 (4.45)	0.95	34	0.35	0.19
MMSE^a^		28 (1.48)	28 (1.48)	0.76	33.96	0.45	0.20
IFS^a^		22 (1.48)	24 (1.48)	3.92	33.98	<0.001***	0.72
**Semantic memory tasks**							
Picture naming^a^	LT	23 (1.48)	23 (0.00)	0.95	28.28	0.35	0.26
	NLT	22 (1.48)	24 (0.00)	6.71	17	<0.001***	0.9
	Tools	11 (0.48)	12 (0)	8.95	17	<0.001***	0.76
	Non-tools	12 (0)	12 (0)	1.84	17	0.08	–
	Total score	45 (1.48)	47 (0.00)	5.14	22.23	<0.001***	0.66
Naming an oral description^a^	LT	21 (2.97)	22 (2.97)	1.77	32.45	0.09	0.38
	NLT	21.50 (2.22)	23.50 (0.74)	2.92	33.86	0.006**	0.46
	Tools	11 (1.48)	12 (0)	3.38	22.56	0.003**	0.69
	Non-tools	11 (1.48)	12 (0)	0.77	33.9	0.45	0.15
	Total score	43 (4.45)	45 (2.97)	2.31	28.66	0.03*	0.43
Word-picture matching^a^	LT	24 (0)	24 (0)	–^c^			
	NLT	24 (0)	24 (0)	1.16	17	0.26	–
	Tools	12 (0)	12 (0)	1.16	17	0.26	–
	Non-tools	12 (0)	12 (0)	–^c^			
	Total score	48 (0)	48 (0)	1.51	17	0.15	–
KDT^a^		48 (2.97)	50 (1.48)	2.13	25.85	0.04*	0.5
PPT^a^		50 (1.48)	51 (1.48)	1.25	34	0.22	0.21
**Lexical fluency task**							
Semantic category^a^	Body parts	20 (4.45)	24 (5.93)	2.12	27.8	0.04*	0.43
	Clothes	14 (2.97)	18.50 (5.19)	3.08	26.85	0.005**	0.56
	Parts of the house	15 (5.93)	20 (7.41)	1.95	30.7	0.06	0.36
	Furniture	10.50 (5.19)	11.50 (3.71)	0.27	28.77	0.79	0.05
	Food and drink	19 (5.93)	22 (7.41)	0.86	33.5	0.40	0.17
	Kitchen	16 (7.41)	16 (5.93)	0.16	33.08	0.87	0.03
	Town	14 (3.71)	17 (6.67)	1.49	34	0.14	0.29
	Countryside	10.50 (3.71)	12 (4.45)	1.49	33.47	0.15	0.31
	Animals	19 (4.45)	22 (5.93)	0.72	28.5	0.48	0.14
	Professions	14 (4.45)	14.50 (5.93)	0.33	30.23	0.75	0.06

Values are expressed as medians and median absolute deviations (MAD). PD, Parkinson’s disease; H&Y, Hoehn and Yahr Scale (Hoehn and Yahr, 1967); UPDRS, Unified Parkinson’s Disease Rating Scale (Fahn and Elton, 1987); GDS, Geriatric Depression Scale (Yesavage et al., 1982); IADL, Lawton Instrumental Activities of Daily Living Scale (Lawton and Brody, 1969); ADL, Barthel Index for Activities of Daily Living (Mahoney and Barthel, 1965); ACE-R, Addenbrooke’s Cognitive Examination Revised (Mioshi et al., 2006); MMSE, Mini Mental State Examination (Folstein et al., 1975); IFS, INECO Frontal Screening battery (Torralva et al., 2009); LT, Living things; NLT, Non-living things; KDT, Kissing and Dancing Test (Bak and Hodges, 2003); PPT, Pyramids and Palm Trees (Howard and Patterson, 1992). ^*a*^*p*-values were calculated through the Yuen’s test (γ). ^*b*^*p*-values were calculated through the chi-squared test (χ^2^). ^*c*^In some cases, Yuen’s test could not be conducted as the difference between medians, or the variance were 0. In those cases, the estimation of effect sizes was also impeded. Significance coding: *p < 0.05; **p < 0.01; ***p < 0.001. Alpha level was set at 0.05 for all analyses.

No subject in any group presented a history of alcohol/drug abuse, physical or psychiatric conditions, or other neurological illnesses. Also, the groups were comparable in terms of their independent living skills and depressive symptoms, as measured with the Lawton Instrumental Activities of Daily Living Scale (IADL) (Lawton and Brody, 1969) and the Barthel Index for Activities of Daily Living (ADL) (Mahoney and Barthel, 1965), and the Geriatric Depression Scale (GDS) (Yesavage et al., 1982; Gomez-Angulo and Campo-Arias, 2011), respectively (see Table 1). All participants provided written informed consent in agreement with the Declaration of Helsinki. The Ethical Research Committee of Universidad del Valle (CIREH 203-015, CI 5278) approved all the study procedures.

### Materials

#### General Cognitive State and Executive Functioning

The participant’s general cognitive state was assessed using the Addenbrooke’s Cognitive Examination Revised (ACE-R) (Sarasola et al., 2005; Mioshi et al., 2006; Reyes et al., 2009), which allows to simultaneously calculate the Mini-Mental State Examination (MMSE) (Folstein et al., 1975) score. This instrument has been extensively used in neurodegenerative diseases (Mioshi et al., 2006; McColgan et al., 2012; Hsieh et al., 2013). The maximum total score in the ACE-R is 100 points (see Supplementary Section 1).

Furthermore, subjects’ executive functioning was examined through the INECO Frontal Screening (IFS) (Torralva et al., 2009), a validated test to measure executive dysfunction in neurodegeneration (Gleichgerrcht et al., 2011; Broche-Pérez et al., 2019; Moreira et al., 2019). This test comprises the following eight subtests: (1) motor programming (Luria series, “fist, edge, palm”); (2) conflicting instructions (hitting the table once when the administrator hits it twice, or hitting it twice when the administrator hits it only once); (3) motor inhibitory control; (4) numerical working memory (backward digit span); (5) verbal working memory (months backward); (6) spatial working memory (modified Corsi tapping test); (7) abstraction capacity (inferring the meaning of proverbs), and (8) verbal inhibitory control (modified Hayling test). The maximum total score in the IFS is 30 points.

#### Semantic Memory Tasks

##### CaGi Battery

The participants performed a previously Spanish adapted version (Moreno-Martínez and Rodríguez-Rojo, 2015; Navarro et al., 2020) of the CaGi battery (Catricalà et al., 2013), which has been widely used in neurodegenerative conditions (Catricalà et al., 2013, 2014, 2015; Della Rosa et al., 2014). This battery includes a set of 48 stimuli belonging to both living (12 animals and 12 vegetables) and non-living entities (12 tools and 12 non-tools).

Specifically, we used the following three subtests: (a) picture naming task, asking the participants to name colored pictures, (b) naming in response to an oral description requiring examinees to name each stimulus after listening to its verbal description (i.e., “*It grows in clusters, has a round shape, is used to make wine.*”), and (c) word-picture matching task, requiring subjects to select, from three pictures, the one corresponding to the spoken word. Correct and incorrect responses were assigned scores of 1 and 0, respectively. Thus, the maximum global score in each task is 48 points.

##### Pyramids and Palms Trees and Kissing and Dancing Tests

The subjects performed the picture version of two additional tasks assessing semantic memory for objects and actions: the Pyramids and Palms Trees test (PPT) (Howard and Patterson, 1992) and the Kissing and Dancing test (KDT) (Bak and Hodges, 2003). Both tests have been previously used in neurodegenerative diseases (Bak et al., 2001, 2006; Ibáñez et al., 2013). In the PPT, participants are shown 52 triplets of object drawings (1 target, 1 correct match semantically related, and 1 distractor non-semantically related) and asked to match the target picture with the one semantically related. The KDT task structure is analogous to the PPT, but stimuli consisted of pictures depicting actions instead of objects. In both tests, one point is earned for each correct answer, resulting in global scores out of 52.

#### Lexical Fluency Measures

LA was measured using 10 semantic categories (SC) of the Pan-Hispanic project (PPHDL available at www.dispolex.com), based on the indications for defining the fundamental lexicon of a language (Sánchez and Aguirre, 1992). SC represented an area related to everyday life, including (1) parts of the body, (2) clothes, (3) parts of the house, (4) furniture, (5) food and drinks, (6) kitchen, (7) town, (8) countryside, (9) animals, and (10) professions. In each SC, the participants were asked to orally generate words for 2 min, avoiding producing proper nouns or repeating words. The participants’ answers were recorded and analyzed offline. One point was assigned for each correct generated word.

### Statistical Analysis

#### Between-Group Comparisons and Statistical Learning Analysis

Normality was evaluated using the Shapiro-Wilk test. Since the assumption of normality was not met, we tried several transformations but none of them normalized the data, so we retained the original scores and proceeded using Yuen (1974)’s test (γ) for between groups comparisons of demographic and behavioral data. Sex was analyzed using the chi-squared test (χ^2^). The statistical significance level was set at *p* < 0.05 for all analyses. Effect sizes were calculated through Wilcox and Tian’ξ (2011), implemented in the WRS2 package (Mair and Wilcox, 2020).

Additionally, statistical learning analyses were conducted to explore which measures best classify groups using the smallest possible set of variables. The predictors were categorized into demographic and neuropsychological (*dem/nepsy*) and lexical (*lex*) clusters. The *Dem/nepsy* cluster included age, years of education, sex, ACE-R, MMSE, IFS, working memory index, the CaGi battery total scores, and the living/non-living subscores, the KDT, and the PPT scores as predictors. The SC of the LA task was introduced as a covariate in this cluster. The *lex* cluster included log-frequency, number of letters, orthographic neighborhood, number of phonemes, number of syllables, familiarity, imageability, and concreteness as predictors.

Then, each cluster of variables was submitted to “one rule” (*1R*) (Holte, 1993) and Boruta (*B*) (Kursa and Rudnicki, 2010) classification algorithms, which rank the variables according to their classification accuracy (*1R*) and relative importance (*B*), respectively. The three strongest classifiers identified by each algorithm were kept.

Finally, four logistic regression models were conducted to ascertain which combination of variables had the highest predicting level (see Table 2). Each model included a combination of two of the strongest classifiers of the *dem/nepsy* and *lex* clusters as independent variables and group (PD patients and controls) as the dependent variable, following the structure *group* ∼ *lex* + *dem/nepsy*. The models were fitted using the standard GLM with a binomial distribution (logit link function). The best classification model was represented via classification trees and spinograms (Everitt and Hothorn, 2014). All analyses were conducted using R version 3.6 (R Core Team., 2020). The R codes and data sets are available at https://figshare.com/projects/memory_and_lexicality_in_Parkinson/99800.

**TABLE 2:** Logistic regression models combining the four variables suggested by the classification algorithms.

	**Metrics**				
**Predictor variables**	***z (p)***	**VIF**	***p-R^2^***	**AIC**	**BIC**
Denomination NLT + familiarity	Denomination NLT = −3.50 (0)	1	0.21	544.25	556.84
	Familiarity = −0.46 (0.65)	1			
Denomination NLT + imageability	Denomination NLT = −3.51 (0)	1	0.21	539.89	552.48
	Imageability = −2.47 (0.01)	1			
IFS total score + familiarity	IFS = −5.98 (0)	1	0.09	623.53	636.11
	Familiarity = −0.64 (0.53)	1			
IFS total score + imageability	IFS = −6.02 (0)	1	0.10	620.21	632.80
	Imageability = −1.71 (0.09)	1			

*Abbreviations: g, group (Parkinson vs. Control); dnlt = denomination of non-living things; t.IFS, total_IFS; im, imageability; fa, familiarity. z (p), z-value and associated *p*-value; VIF, variance inflation factor; p-R^2^, McFadden pseudo-R^2^ (see Table 6 in Hemmert et al., 2018 for interpretation); AIC, Akaike information criterion; BIC, Bayesian information criterion. The model with the best fit is shaded in gray.*

#### Lexical Availability Analysis

##### First Step

All perseverative responses were excluded. We used the lexical statistical program Dispolex (available at http://www.dispolex.com) following previous studies (Samper-Padilla, 1998; Bartol-Hernández and Hernández-Muñoz, 2003; Hernández-Muñoz et al., 2006, 2014; Mateus and Santiago, 2006; López-Morales, 2014). This program provided us: (a) the total number of words’ occurrences (tokens), (b) each lexical unit (types) counts, (c) the average number of responses, and (d) the frequency and position of each word in each semantic category (LA index), and (e) the degree of coincidence in informants’ word response (lexical cohesion index) (Echeverría, 1991; Hernández-Muñoz, 2010).

##### Second Step

In each category, words with a frequency of appearance lower than 4.17% (frequency equal to 1) were excluded. Subsequently, a lexical properties analysis was conducted by identifying: (a) orthographic structure: word frequency and number of letters, (b) orthographic neighborhoods: Levenshtein distance (Levenshtein, 1966), (c) phonological structure: number of phonemes and number of syllables, and (d) word’s subjective ratings: familiarity, imageability, and concreteness.

These linguistic variables for Latin American Spanish were identified in the web interface to Spanish word frequency data and other word properties based on written and subtitle corpora (Duchon et al., 2013) (available at https://www.bcbl.eu/databases/espal/).

## Results

### General Cognitive State

No between-group differences were observed in the ACE-R [γ_(__34__)_ = 0.95, *p* = 0.35, ξ = 0.19] and the MMSE [γ_(__33_._96__)_ = 0.76, *p* = 0.45, ξ = 0.20] total scores. However, PD patients performed lower than controls in the IFS total score [γ_(__33_._98__)_ = 3.92, *p* < 0.001, ξ = 0.72], the digits backward subtest [γ_(__28_._66__)_ = 2.65, *p* = 0.01, ξ = 0.44], the working memory index [γ_(__33_._96__)_ = 2.22, *p* = 0.03, ξ = 0.46], and marginally lower in the verbal inhibitory control subtest [γ_(33.31)_ = 1.76, *p* = 0.09, ξ = 0.38] (see Table 1 and Supplementary Table 1).

### Semantic Memory Tasks

#### CaGi Battery

##### Picture Naming Task

PD patients globally scored lower than controls [γ_(__22_._23__)_ = 5.14, *p* < 0.001, ξ = 0.66]. Specifically, patients performed lower than controls in naming non-living things [γ_(__17__)_ = 6.71, *p* < 0.001, ξ = 0.9] and tools [γ_(__17__)_ = 8.95, *p* < 0.001, ξ = 0.76]. No significant between-group differences were observed in the denomination of living things [γ_(__28_._28__)_ = 0.95, *p* = 0.35, ξ = 0.26] and non-tools [γ_(__17__)_ = 1.84, *p* = 0.08] (see Table 1).

##### Naming in Response to an Oral Description

PD patients globally performed lower than controls [γ_(__28_._66__)_ = 2.31, *p* = 0.03, ξ = 0.43]. Particularly, patients exhibited lower scores in naming non-living things [γ_(__33_._86__)_ = 2.92, *p* = 0.006, ξ = 0.46] and tools [γ_(__22_._56__)_ = 3.38, *p* = 0.003, ξ = 0.69]. The groups’ performance did not differ in naming living things [γ_(__32_._45__)_ = 1.77, *p* = 0.09, ξ = 0.38] and non-tools [γ_(__33_._9__)_ = 0.77, *p* = 0.45, ξ = 0.15] (see Table 1).

##### Word-Picture Matching

No significant differences between groups were observed in the global performance [γ_(__17__)_ = 1.51, *p* = 0.15], and the denomination of living things (equal medians), non-living [γ_(__17__)_ = 1.16; *p* = 0.26], tools [γ_(__17__)_ = 1.16, *p* = 0.26] and non-tools categories (equal medians) (see Table 1).

#### Pyramids and Palms Trees and Kissing and Dancing Tests

KDT total score was lower in PD patients than in controls [γ_(__25_._85__)_ = 2.13, *p* = 0.04, ξ = 0.5], there being no significant between-group differences in the PPT scores [γ_(__34__)_ = 1.25, *p* = 0.22, ξ = 0.21] (see Table 1).

### Lexical Fluency Performance

Qualitatively, PD patients exhibited a lower total number of words (tokens) in large (i.e., countryside) and small (i.e., parts of the body) semantic categories (see Supplementary Section 2.1 and Supplementary Table 3).

#### Lexical Units Index

In PD patients, the two SC with the most different lexical units corresponded to animals (79 lexical units) and food and drinks (74 lexical units). In contrast, the least productive SC were countryside (33 lexical units) and furniture (38 lexical units). In Supplementary Table 3, there was no direct relationship between general lexical productivity and word types (a measure of lexical richness).

In controls, the most productive SC with the highest number of word types were food and drinks (83 lexical units) and body parts (74 lexical units). Like the PD group, the least productive SC were countryside (42 tokens) and furniture (43 lexical units).

#### Lexical Availability Index and Lexical Cohesion Index

Results are summarized in Supplementary Section 2.2, 2.3 and Supplementary Tables 1, 2.

### Statistical Learning Analysis

In the *dem/nepsy* cluster, the denomination of non-living things, the global denomination score, and the total IFS score were the strongest variables for distinguishing between groups, correctly classifying 79.6% (58.3% of PD and 100% of controls), 77.5% (54.2% of PD patients and 100% of controls), and 69.4% (75% of PD patients and 64% of controls) of the overall cases, respectively. These variables also obtained the highest relative importance, only slightly varying in their order: denomination of non-living things (*B* = 26.54), total IFS score (*B* = 22.12), and global denomination score (*B* = 21.74) (see Table 3).

**TABLE 3:** Results of the classification accuracies and variable’s importance.

**Variable cluster**	**Variable**	**Algorithm (ranks)**	
		***1R* Classification accuracy (%)^a^**	***B* Relative importance^b^**
Demographic and neuropsychological *(dem/nepsy)*	Denomination of non-living things	79.59% (1)	26.54 (1)
	IFS total score	69.39 (3)	22.12 (2)
	Global denomination score	77.55% (2)	21.74 (3)
Lexical *(lex)*	Imageability	53.48% (2)	0.69 (3)
	Familiarity	54.49% (1)	0.75 (2)
	Levenshtein distance	52.65% (3)	
	Concreteness		1.71 (1)

*The best three variables per classification algorithm are shown. ^*a*^Values calculated through the one-rule (*1R)* algorithm. ^*b*^Values calculated through the Boruta (*B)* algorithm. In the case of the *Lex* variables, the *B* algorithm indicated that none of the variables was deemed necessary (see details in the Supplementary Material). Note that in the case of *dem/nepsy* variables, all three variables were common to both classification algorithms, and while *denomination of non-living things* was the best according to each algorithm, *IFS* total score and global denomination *score* were equally valid; for simplicity though one of these was retained for further analyses. All variables retained for further analyses are shaded in gray. Empty cells are cases when the variables *Levenshtein distance* and *concreteness* had ranks above three and/or gave classification accuracies below 50%.*

In the *lex* cluster, familiarity, imageability, and Levenshtein distance were the strongest predictors of group membership, successfully classifying 55% (58.8% of PD patients and 50% of controls), 53.5% (60% of PD patients and 48% of controls), and 52.7% (12.1% of PD patients and 91.6% of controls of the total cases, respectively. Besides, concreteness reached the highest relative importance (*B* = 1.71), followed by familiarity (*B* = 0.75) and imageability (*B* = 0.69) (see Table 3). Nevertheless, both classification algorithms indicated that these and other *lex* variables had classification accuracies near chance (*1R*) and low importance (*B*) (see Table 3).

### Logistic Models

The model combining the denomination of non-living things (*z* = −3.51, *p* < 0.01) and imageability (*z* = −2.47, *p* = 0.01) reached the best fit (*p-R^2^* = 0.21, AIC = 539.89, BIC = 552.48) (see Table 3). However, this model was not pursued given the results of the classification algorithms regarding the *lex* variables; as shown in Table 1, all lexical variables had classification accuracies near chance (*1R* algorithm) and very low importance (*B* algorithm). Thus, the model *group* ∼ *dnlt* was examined via a classification tree and a spinogram.

**FIGURE 1:**
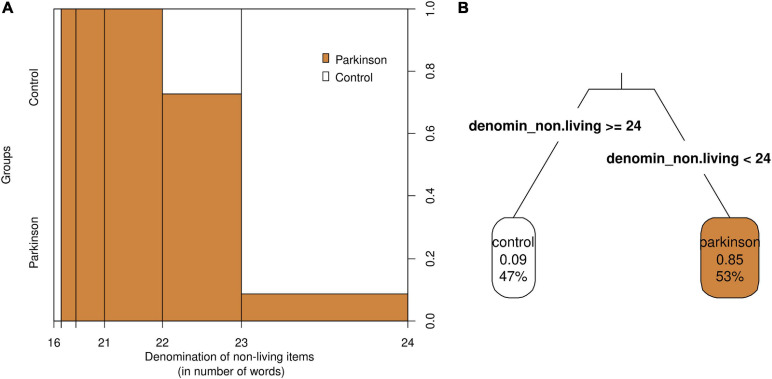
Spinogram **(A)** and classification tree **(B)** of the model *group* ∼ *dnlt*. **(A)** The widths of the bins in the *x*-axis in the spinogram represent the frequencies (number of participants that obtained a score) within each bin. For example, there were more observations between 23 and 24 denominations than between 16 and 21 denominations. Colors represent groups, white being for controls (always on top) and orange being for PD patients (always below). The right y-axis represents the proportion of subjects that belonged to each group in each of the bins. **(B)** The classification tree shows the likelihood of being classified as control or PD depending on a cut-off score of 24 in the denomination of non-living things subtask.

The classification tree results suggested that when a person produces less than 24 denominations of non-living things, there is about an 85% chance of being classified as a PD patient. If the person produces about 24 or more denominations, the chances of the person being classified as a PD patient are about 9% (Figure 1B). The spinogram further corroborates these approximate likelihoods and provides the observed counts for different bins (Figure 1A). It is important to stress that the cut-offs are merely approximations and need to be revised within the task context.

## Discussion

This study aimed to characterize the cognitive performance of PD patients using a comprehensive set of lexical fluency and SM tasks and determine the smallest set of measures that best classify the groups. The classification algorithms indicated that some of the SM tasks had the highest classification accuracies while none of the executive or lexical variables had reliably classified groups. In particular, the “denomination of non-living things” had the highest classification accuracy of ∼80%.

### Semantic Memory in PD

PD patients showed an inferior performance in two naming tasks of CaGi measures. In line with previous studies, significant differences were observed in the visual and auditory input tasks (Portin et al., 2000; Rosenthal et al., 2017; Salmazo-Silva et al., 2017). Importantly, this inferior performance was most notable in the SM category of non-living things.

From an embodied perspective (Tirado et al., 2018; Khatin-Zadeh et al., 2021), these results could be attributed to PD patients’ difficulty to access manipulable objects’ semantic representation. Previous studies suggest that PD is associated with deficits in the semantic representation of actions/verbs that imply movement (Cardona et al., 2014; Bocanegra et al., 2015; Melloni et al., 2015; Suárez-García et al., 2021) or functional manipulability (Péran et al., 2009; Herrera et al., 2012; Bocanegra et al., 2017). This poor PD performance is associated with the disrupting basal ganglia-frontal circuit activated during action processing and object manipulation tasks. It has been shown that this circuit participates in the crucial coupling between motor and linguistic information (Pulvermüller, 2005; Pulvermüller et al., 2005; Melloni et al., 2015) and that its disruption hinders such coupling (Ibáñez et al., 2013). However, as this study did not include neurophysiological/neuroimaging measures, further evidence is needed to support this view. As the semantics of manipulable objects entails body movement, deterioration of the mentioned circuit might explain why PD patients have a challenging time accessing these semantic representations. This is further confirmed by the findings in the tools’ subcategory of the picture naming and naming on oral description tasks, in contrast to the non-tools subcategory (although there was a trend in the first task). These results converge with a growing corpus of research showing impairments in action semantics in PD and hint that the possibility of impairments in the semantic processing of non-living things is likely to be driven by the presence of motor representations (manipulability) in the semantic store of these objects.

As previous research has shown, manipulable objects naming is particularly impaired in PD (Johari et al., 2019). However, it might be possible to account for these deficits with techniques such as subthalamic DBS even in early PD (Phillips et al., 2012). The present findings also suggest that the comprehension of manipulable objects might deteriorate, so its treatment should also be explored through adjuvant electrical stimulation techniques.

Although PD patients did not present mild cognitive impairment, EF deficits were observed, especially in working memory and partially in verbal inhibitory control, as measured in the IFS scale by the digits backward task, and a shortened version of the Hayling test, respectively. These results agree with previous studies highlighting executive dysfunction as a frequent trait in PD’s initial stage (Barone et al., 2011; Khoo et al., 2013; Liu et al., 2017). Furthermore, while the IFS global score reached a high classification accuracy, it was not superior to that of denomination of non-living things, hinting that these semantic deficits might be more characteristic to PD than executive deficits.

### Lexical Availability in PD

Meta-analysis has shown that non-demented PD patients have semantic fluency impairments (Henry and Crawford, 2004; Kudlicka et al., 2011). Some authors suggest a selective lexical retrieval impairment in PD and frontal patients (Rogers et al., 1998; Silveri et al., 2017; Johari et al., 2019). Tagini et al. (2018) speculate that this deterioration may be due to a low activation level (difficulty in initiation, bradyphrenia) that slows down the production rate throughout the task or a damaged semantic store.

No previous research has explored the lexical availability in PD. Our study’s total number of words per semantic field was inferior in the PD group in both large and small semantic categories. These results indicate that PD patients present an overall more deficient search strategy in the semantic store and deficits in switching from one subcategory to another than controls. The inferior performance shown in these semantic categories is expectable given the delay of speech initiation, bradyphrenia, and the fact that PD patients perform worse than healthy controls in all categories, although not all of them reached statistical significance.

Semantic fluency tasks are less automatic than naming or matching tasks (Fernandino et al., 2013; Salmazo-Silva et al., 2017). Several cognitive domains contribute to performance on fluency tasks (Rosen and Engle, 1997; Reverberi et al., 2006, 2014; Unsworth et al., 2011; Robinson et al., 2012; Tagini et al., 2018). In this way, generating search strategies and concepts’ internal organization is critical for satisfactory performance.

### Limitations

This work has significant limitations. First, we did not use the complete CaGi battery, including the picture sorting, free generation of features, and sentence verification subtests due to the participants’ fatigue and/or disinterest. Another limitation is the absence of the switching and clustering index. Without these analyses, semantic proximity is unknown, and therefore, it cannot be inferred whether the observed deficits are associated with alterations in strategic retrieval processing or monitoring deficits. These limitations prevent a broader interpretation of the results. Finally, we acknowledge that the levodopa equivalent dose is a highly relevant variable missing in this study since previous studies have shown an effect of dopaminergic medication in semantic processing related to action (Boulenger et al., 2008; De Letter et al., 2012, 2020).

## Conclusion

To summarize, our results suggest that semantic memory is affected in early-stage non-demented PD patients. More importantly, a potential dissociation between living and non-living things categories was found, consistent with previous findings in the study of cognition in PD and the embodied perspective of cognition. Future studies involving neuroimaging techniques can provide fine-grained spatial and functional brain information.

## Data Availability Statement

The datasets presented in this study can be found in online repositories. The names of the repository/repositories and accession number(s) can be found in the article/Supplementary Material.

## Ethics Statement

The studies involving human participants were reviewed and approved by the Ethical Research Committee of Universidad del Valle (CIREH 203-015, CI 5278). The patients/participants provided their written informed consent to participate in this study.

## Author Contributions

JFC, GM-F, and FM-R developed the study concept and the study design. JG-C, CT-L, LT, JC, JD, and TJ performed the testing and data collection. JFC, FM-R, JG-C, CT-L, and GM-F performed the data analysis and interpretation. JFC, JG-C, CT-L, HU, SC, AT, LG, and JC drafted the manuscript. NO-C, FM-R, and GM-F provided the critical revisions. All authors contributed to the article and approved the submitted version.

## Conflict of Interest

The authors declare that the research was conducted in the absence of any commercial or financial relationships that could be construed as a potential conflict of interest.

## Publisher’s Note

All claims expressed in this article are solely those of the authors and do not necessarily represent those of their affiliated organizations, or those of the publisher, the editors and the reviewers. Any product that may be evaluated in this article, or claim that may be made by its manufacturer, is not guaranteed or endorsed by the publisher.
