# Supplementary material for: Semantic Memory and Lexical Availability in Parkinson’s Disease: A Statistical Learning Study
Source: Front Aging Neurosci. 2021 Jul 30;13:697065. doi: 10.3389/fnagi.2021.697065 (PMC8361833; doi:10.3389/fnagi.2021.697065)
Supplement: Supplementary file 1 [file Data_Sheet_1.docx]

**Supplementary material**

**Semantic memory and lexical availability in Parkinson’s disease: A statistical learning study**

**S1. Cognitive assessment**

The Addenbrooke´s Cognitive Examination-Revised (ACE-R, Torralva et al, 2011) is a brief and sensitive test for the early stages of cognitive decline in neurodegenerative disorders, including Alzheimer's disease, frontotemporal dementia, progressive supranuclear palsy, Parkinson’s disease (Chade et al., 2008; Reyes et al., 2009) and other motor disorders (Bak et al., 2005). The ACE-R comprises orientation, attention, memory, language, visuoconstructional/visuospatial skills, conceptual thinking and calculations tasks.

**S2. Lexical availability analysis**

**S2.1 General lexical productivity (tokens)**

***Parkinson’s disease group***

In PD group, the total amount of tokens was 2673. The three more productive SF were body parts (428 tokens), animals (391 tokens), and food and drinks (341 tokens). Conversely, the least productive tokens were countryside (94 tokens) and furniture (185 tokens). These productivity differences between SF could be linked to each SF conceptual familiarity or semantic relevance.

***Control group***

The group’s total number of tokens was 3200. The three most productive SF included body parts (502 tokens), food and drinks (409 tokens) and animals (403 tokens). Also, similar to PD group, the least productive SF were countryside (150 tokens) and furniture (227 tokens).

**S2.2 Lexical availability index (LAI)**

Lexical availability accounts for a set of lexical units ordered by frequency and place on the list in each SF. Below are presented the results from five representative SF:

***Parkinson disease patients***

In body parts, the most available word was *nose*, with a LAI : 0.81, being the first word evoked in 95.83% of PD patients, followed by *eye* (LAI : 0.71; 87.5%), and *finger* (LAI :0.70; 91.67%). In the SC clothes, the most available word was *shirt* (LAI : 0.85; 95.83%) followed by *pant* (LAI : 0.58; 62.5%) and shoe (LAI : 0.55; 83.3%). In the SC animals, we find first, the word *cat* with a LAI : 0.67 (87.5%), followed by dog (LAI : 0.64; 75%) and horse (LAI : 0.50; 66.7%). In foods and drinks, we found rice (LAI : 0.38; 54.17%); juice (LAI : 0.37; 50%) and wine (LAI : 0.35; 45.83%). Regarding furniture, we found dining room (LAI : 0.44; 58.3%), couch (LAI : 0.39; 54.17%) and bed (LAI : 0.38; 62.50%).

***Control group***

Regarding the control group, in parts of the body, the most available word was head (LAI: 0.71), being the first word evoked in 84.0% of control subjects, followed by eye (LAI : 0.69; 88%) and finger (LAI : 0.57; 76.0%). In the SC clothes, the most available word was pant (LAI : 0.74; 84.0%), sock (LAI : 0.70; 70%) and shirt (LAI : 0.62; 80%). In the SC animals, we found first the word dog with a LAI : 0.73 (84.0%), followed by cat (LAI : 0.64; 76.0%) and horse (LAI : 0.45; 68.0%). In foods and drinks, we found rice (LAI : 0.61; 76.0 %), meat (LAI :0.43; 60%), potato (LA : 0.40; 56.0%). Regarding furniture, we found bed (LAI : 0.57; 84.0%), chair (LAI : 0.54; 64.0%) and coach (LAI :0.41; 52%).

**S2.3 Lexical cohesion index (LCI)**

This index is calculated by dividing the average of responses of each subject by the LUI in each SC, which accounts for the dispersion or coincidence in each SC's responses. In this regard, a score near 1 indicates a more compact (or closer) SC, whereas if it moves away from that reference point, the SC is considered fuzzy or open.

***PD patients***

The two SC with the highest LCI were parts of the body (0.31) and clothing (0.24). By contrast, the most diffuse SCs were countryside (LCI : 0.12) and professions (0.15). These differences in productivity between semantic fields are associated with conceptual familiarity and semantic relevance.

***Control group***

The SC with the highest LCI corresponds to parts of the body (0.27) and clothing (0.24). Like the PD group, the most diffuse or open SCs were countryside (LCI : 0.14) and professions (0.14).

**Supplementary tables**

**Supplementary Table 1. INECO Frontal Screening battery subscores**

|  | PD patients  (*n*:30)  *Median (±MAD)* | Controls  (*n*:30)  *Median (±MAD)* | PD vs. controls | | | |
| --- | --- | --- | --- | --- | --- | --- |
|  |  |  | *γ* | *df* | *p* | *ES* |
| Motor programming^a^ | 3 (0) | 3 (0) | 0.57 | 33.78 | 0.57 | 0.11 |
| Conflictive instructions^a^ | 3 (0) | 3 (0) | 0 | 34 | 1 | 0 |
| Go-No-Go^a^ | 3 (0) | 3 (0) | 0.51 | 33.97 | 0.61 | 0.1 |
| Digits backward^a^ | 3 (1.48) | 3 (1.48) | 2.65 | 28.66 | 0.01^**^ | 0.44 |
| Verbal working memory^a^ | 2 (0) | 2 (0) | -^b^ | | | |
| Spatial working memory ^a^ | 3 (1.48) | 3 (1.48) | 0.98 | 33.96 | 0.33 | 0.19 |
| Abstraction capacity^a^ | 3 (0) | 3 (0) | 0.28 | 33.96 | 0.78 | 0.06 |
| Verbal inhibitory control^a^ | 4 (1.48) | 6 (0) | 1.76 | 33.31 | 0.09 | 0.38 |
| Working memory index^a^ | 6 (1.48) | 6 (1.48) | 2.22 | 33.96 | 0.03^**^ | 0.46 |
| Total score ^a^ | 22 (1.48) | 24 (1.48) | 3.92 | 33.98 | <0.001^***^ | 3.92 |

Values are expressed as medians and median absolute deviations (MAD)

PD: Parkinson’s disease

^a^ *p*-values were calculated through Yuen’s test (γ)

^b^ In some cases, the Yuen’s test could not be conducted as the difference between medians or the variance were 0. In those cases, the estimation of effect sizes was also impeded.

Significance coding: ^*^ *p*<0.05; ^**^*p*<0.01; ^***^ *p*<0.001

Alpha level was set at 0.05 for all analyses.

**Supplementary Table 2. First lexical unit distributed per prompt and group in each semantic category**

|  | **PD patients** | | | **Controls** | |
| --- | --- | --- | --- | --- | --- |
| **Ssemantic category** | **1^st^ LU** | **LAI** | **1^st^ LU** | | **LAI** |
| Body parts | Nariz (nose) | 0.806 | Cabeza  (head) | | 0.707 |
| Clothes | Camisa (shirt) | 0.852 | Pantalón (pants) | | 0.737 |
| Parts of the house | Sala (living room) | 0.644 | Cocina (kitchen) | | 0.674 |
| Furniture | Comedor (dining room) | 0.449 | Cama (bed) | | 0.571 |
| Food and drink | Arroz (rice) | 0.384 | Arroz (rice) | | 0.611 |
| Kitchen | Estufa (stove) | 0.612 | Olla (cooking pot) | | 0.659 |
| Town | Calle (street) | 0.557 | Edificio (building) | | 0.518 |
| Countryside | Sembrar (sow) | 0.240 | Sembrar (sow) | | 0.393 |
| Animals | Gato (cat) | 0.669 | Perro (dog) | | 0.728 |
| Professions | Médico (physician) | 0.777 | Ingeniero (engineer) | | 0.516 |

LU: Lexical unit; LAI: lexical availability index.

**Supplementary Table 3. Total number of different words per semantic category and number of words per participant**

|  |  | **PD** | **Controls** |  | **PD** | **Controls** |  | **PD** | **Controls** |
| --- | --- | --- | --- | --- | --- | --- | --- | --- | --- |
| **Semantic category** |  | **Word tokens** | |  | **Word types (LU)** | |  | **LCI** | |
| Body parts |  | 428 | 502 |  | 57 | 75 |  | 0.31 | 0.27 |
| Clothes |  | 302 | 367 |  | 52 | 61 |  | 0.24 | 0.24 |
| Parts of the house |  | 270 | 330 |  | 57 | 66 |  | 0.20 | 0.20 |
| Furniture |  | 185 | 227 |  | 38 | 43 |  | 0.20 | 0.21 |
| Food and drink |  | 341 | 409 |  | 74 | 83 |  | 0.19 | 0.20 |
| Kitchen |  | 236 | 301 |  | 45 | 61 |  | 0.22 | 0.20 |
| Town |  | 221 | 272 |  | 47 | 67 |  | 0.20 | 0.16 |
| Countryside |  | 94 | 150 |  | 33 | 42 |  | 0.12 | 0.14 |
| Animals |  | 391 | 403 |  | 79 | 74 |  | 0.21 | 0.22 |
| Professions |  | 204 | 239 |  | 55 | 68 |  | 0.15 | 0.14 |

LU: Lexical units; LCI: Lexical cohesion index.
